# Job Security and Organizational Citizenship Behaviors in Chinese Hybrid Employment Context: Organizational Identification Versus Psychological Contract Breach Perspective Differences Across Employment Status

**DOI:** 10.3389/fpsyg.2021.627934

**Published:** 2021-02-24

**Authors:** Wenzhu Lu, Xiaolang Liu, Shanshi Liu, Chuanyan Qin

**Affiliations:** South China University of Technology, School of Business Administration, Guangzhou, China

**Keywords:** job security, stress theory, counterproductive work behavior, turnover intention, employment status

## Abstract

The goal of the present research was to identify the mechanism through which job security exerts its different effects on organizational citizenship behaviors (OCB) among contract and permanent employees from social identity and social exchange perspectives. Our research suggests two distinct, yet related explanatory mechanisms: organizational identification and psychological contract breach, to extend the job security literature by examining whether psychological contract breach and organization identity complement each other and explaining the mechanism of different behaviors response to job security across employment status. Data were collected from 211 Chinese employees and 61 supervisory ratings of OCBs. Our results showed that relative to psychological contract breach, organizational identification plays a stronger mediating role in the association between job security and OCBs. Evidence from multi-group analyses also suggested employment status moderated the mediation mechanism of organizational identification between job security and OCB. Implications for job security and hybrid employment management are discussed.

## Introduction

Low perception of job security reflects concerns about the continuity of future employment or the threat of losing a current job, which would bring various reaction into an employee’s work, life, and health (Sverke et al., 2002; Hans, 2005; Lam et al., 2015; Shoss, 2017). High levels of job security can create substantial benefits to organizations and employees (Greenhalgh and Rosenblatt, 1984; Hans, 2005; Stynen et al., 2013; Shoss, 2017; Vuuren et al., 2020). However, low levels of job security have become a sizeable social phenomenon, caused by fundamental changes in the economic system of China, such as increase in contractor, technological innovations, and economic downturn (Jiang et al., 2020). For example, previous research demonstrated that the use of contingent workers threatened permanent employees job security because permanent employees may attribute the use of temporary workers to management intentions to change internal structures (Kraimer et al., 2005). Meanwhile, due to the limited employment period of contract workers, they are also at a lower level of job security. Consequently, studies have begun to focus on providing practical suggestions for curbing the negative results of low job security (Hans, 2005; Piccoli et al., 2017; Jiang et al., 2020; Kim et al., 2020; Wang et al., 2020). This burgeoning research has demonstrated that employees’ undesired behaviors, such as low job performance or organizational citizenship behaviors (OCB), may be a result of low job security (Hans, 2005; Haijiang et al., 2014; Ma et al., 2015; Stynen et al., 2013; Callea et al., 2016).

Despite the progress made in the literature in examining the effect of low levels of job security, notable omissions remain. First, research on why job security influences individuals’ discretionary behaviors is scarce and has mainly focused on one explanation, neglecting the possible interplay of manifold mediators in the same relationship (De Witte et al., 2016). Previous research mainly based on stress theory or social exchange theory to explore its effect on individuals’ attitudes and behaviors (Reisel et al., 2014; Lee and Jeong, 2017), and few researches compared the different mediating mechanism between job security and OCB. This is a noteworthy gap, and explicitly testing competing models can sharpen our theoretical understanding of job security.

A second important gap in the extant literature relates to the examination of the difference association between job security and discretionary behaviors among contract and permanent worker in Chinese hybrid employment context. Increased competition prompts organizations to increasingly opt for contract employment relations with their employees. Hybrid employment in organizations is becoming a prevailing phenomenon (Johnson and Ashforth, 2008; Cappelli and Keller, 2013; De Jong et al., 2018; Stefano et al., 2018), which questions whether the effects of job security on discretionary behaviors are different across employment status, and begs examining why. Although previous research has demonstrated that the relationship between job security and OCBs could be different across employment status (Liu et al., 2017), it remains unclear why job security motivates contract employees to engage in more or less OCB compared to permanent employees. It is noteworthy that Witte and Naswall (2003)’s research indicates that job security includes objective and subjective phenomenon, and contract workers typically display lower objective job security than permanent worker due to their poor job conditions. However, research also demonstrated that irrespective of the objective situation, workers might perceive different levels of job security (Klandermans et al., 2010). In our research, we focused on subjective job security among contract and permanent worker.

These are important omissions in the literature, and addressing these problems can further our understanding of the mechanisms by which job security exerts its effects in hybrid employment contexts. Therefore, our research aimed to extend the job security literature by providing multiple explanatory mechanisms for the different discretionary behaviors associated with job security across employment status. In this study, we aim to increase the understanding of the effects of job security on discretionary behaviors, that is, OCB. OCB, defined as individuals’ discretionary behaviors that go beyond formal job descriptions and contribute to the organization’s success, are part of contextual performance and are critical for organization (Organ, 1997). Therefore, in order to prompt employees’ OCB, it is important to understand how and why job security may motivate employees’ OCB and to suggest theoretical explanations for these relationships.

Our aim was to explain the relationship between job security and outcomes across employment status by two distinct, yet related mechanisms: psychological contract breach and organizational identification. From the perspective of psychological contract theory, psychological contract refers to an implicit, unwritten agreement between parties to respect each other’s norms, which contain mutual obligations between organization and employees (Robinson et al., 1994). One of its fundamental principles is the balance between employees’ investment and outcomes (Rousseau and Tijoriwala, 1998; Piccoli, and De Witte, 2015). Contract employees who sign a limited contract with an organization perceive fewer and a narrower range of employer obligations (Broschak and Davis-Blake, 2006; Cappelli and Keller, 2013). This imply they would be less likely to believe that the employer is obligated to secure their jobs, compared to employees with permanent positions. By contrast, the content of the psychological contract of permanent employees includes a broader array of employer’s obligations and with a long-term focus (De Cuyper et al., 2009). Accordingly, permanent employees may interpret low levels of job security relatively more than contract worker as a breach of the psychological contract. Extending this to the employees’ obligation to perform OCBs, we would expect that contract employees would be more likely to engage in OCBs than permanent worker when they experience psychological contract breach related with low levels of job security.

**FIGURE 1:**
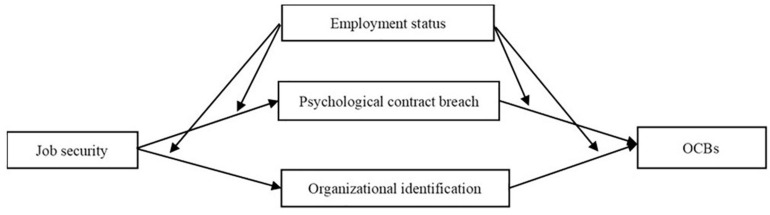
Conceptual model.

Another perspective that explains the different effects of job security on OCB across employment status is expressed by the social identity theory (Riketta, 2005; Ashforth et al., 2008). Organizational identification refers to a “part of an individual’s self-concept that derives from his knowledge of his membership of a social group (or groups) together with the value and emotional significance attached to that membership” (Ashforth et al., 2008). Social identity perspective emphasizes the motivation of employees’ self-enhancement and uncertainty reduction by seeking organizational identification. This perspective argues that individuals will use the status of their organization to assist them in assessing their self-worth (Fuller et al., 2016). In the hybrid employment context, contract employees are usually associated with the organization’s periphery, which, in turn, has been related with inferior job characteristic and lower organization status (Stamper and Masterson, 2002). At the same time, they are confronted with low levels of job security. Therefore, the contract employees’ urge of seeking organizational identification is stronger than permanent employees, due to their poor job situation. The research model is shown in Figure 1.

Our investigation contributes to the job security literature in three major aspects. First, we extend the knowledge on the theoretical explanations of the positive outcomes associated with job security by integrating the two previously presented mechanisms of organizational identification and psychological contract breach in hybrid employment contexts. We build on the social identity and psychological contract breach perspective to make predictions on the relationship between job security and OCB (Rousseau and Tijoriwala, 1998; Riketta, 2005; Fuller et al., 2016). Second, we help to clarify the relative importance of psychological contract breach and organizational identity mechanisms with respect to the association between job security and OCB. We also contribute to the job security literature. Third, by exploring why contract employees engage in more or less OCBs than permanent employees from the perspective of organizational identification and physical contract breach. This enables examining which of psychological contract breach and organizational identity plays a more leading role in accounting for the different levels of OCB related to job security across employment status by using a two-phase survey design, which broadens the research related to job security.

## Theory and Hypothesis Development

### Job Security and OCBs

According to the social exchange theory, the norm of reciprocity indicates that when organizations treat employees in a positive manner, employees should respond favorably by engaging in positive behaviors, such as OCBs (Cropanzano and Mitchell, 2005; Lam et al., 2015). Thus, in our research, we assumed that job security is positively related with OCB. Employees usually consider a secured employment as part of their implied contract with the employer, and expect that the organization will reciprocate by offering them rewards in terms of job security when they work hard (Piccoli et al., 2017). Whereas, employees under the threat of a job loss may perceive the unbalanced exchange relationship between their investment and outcomes they received, which would weaken their motivation to reciprocate the organization in terms of OCBs. The imbalanced exchange relationship resulting from a low job security damages the reciprocity principle of social exchange theory, and employees may restore the balance of the exchange relationship with the organization by reducing their OCBs. However, employees with a high job security tend to view it as a benefit from the organization; they are more likely to establish trust and mutual care relationships with the organization, thus, performing more OCBs in return for concerns and helps from colleagues. There are also numerous empirical researches that demonstrate job security is positively related to OCB. For example, research used a multiple-group path analysis of age groups to show that qualitative job insecurity can frustrates people’s intrinsically motivated OCB (Stynen et al., 2013). Ma et al. (2015) also demonstrated that job insecurity is negatively related with OCB in the Chinese context (Ma et al., 2015). Thus, we hypothesize that:

H1. Job security is positively related to OCBs.

### Mediating Effects of Psychological Contract Breach

Job security, as part of a high-quality exchange relationship, is expected to exert a negative influence on employees’ psychological contract breach. Psychological contract has been mainly used as a framework that captures the implicitness of the exchange relationship between an employee and their employer. Reciprocity is a critical foundation to explaining psychological contract breach. One psychological contract may be the provision of hard work and effort by employees in exchange for a secured employment at the organization (Cuyper and Witte, 2006; Probst et al., 2018). When job security declines, employee perceives their employer has not fulfilled their obligations and a breach of the moral norm of reciprocity, which lead to a psychological contract breach. Existing studies have also shown that a low level of job security could lead to employees’ perception of psychological contract breach (Cuyper and Witte, 2006).

Furthermore, employees react to treatments received from organization based on the social exchange theory and principle of reciprocity (Cropanzano and Mitchell, 2005). On the one hand, OCB may be an indicator of favorable exchange relationship with organization. However, psychological contract breach indicates the low quality of employee-organization exchange relationship. According to social exchange theory, employees may decrease their OCBs when they perceive a psychological contract breach based on the reciprocity principle. The implicit assumption is that employees’ judgment regarding whether their organization have kept their contract terms would be a motivation for performing OCBs. On the other hand, research also considers a psychological contract breach an emotional manifestation of a broken promise, accompanied by feelings of betrayal, letdown, disappointment, frustration, and resentment (Hill et al., 2009), which will distract their resources from performing OCBs. Psychological contract breach is reportedly a vital antecedent of employees’ attitudes and behavior in the workplace (a) through the mechanism of depressive mood states (Priesemuth and Taylor, 2016) and (b) by depleting and draining employees, making them absorbed in conserving any remaining resources (Rai and Agarwal, 2017). Hence, low levels of job security could lead to an imbalanced perception between efforts and rewards, which would trigger employees’ perception of psychological contract breach and cut down their OCBs retaliation to the organization. We propose that:

H2. Psychological contract breach mediates the relationship between job security and OCBs.

### Mediating Effects of Organizational Identification

Drawing on social identity theory, employees may return the benefits they receive from their organization in terms of organizational identification (Cropanzano and Mitchell, 2005; Ashforth et al., 2008). This implies that employees are more likely to identify with their organization when they are appreciated by the organization, whereas if employees perceive unfavorable organizational treatments, such as low job security, they may have a low level of organizational identification (Fuller et al., 2016). When employees perceive that the organization cares about their value and provides them with a stable job, they are likely to perceive themselves as insiders and appreciate the organization, which foster identification with the organization (Stamper and Masterson, 2002). In contrast, a low job security is likely to undermine employees’ judgment of their value in the organization, and results in lower levels of organizational identification (Ashforth et al., 2008). Furthermore, organizational identification serves to fulfill the belongingness need (Wu et al., 2016). Low levels of job security threaten employees’ perceived stability and continuance with the organization and hinders the satisfaction of belongingness. Employees under job threatening may perceive that they are no longer a member of the organization, which could jeopardize employee’s ability to launch a self-categorization, generate affiliation, and establish self-concept in the organization (Riketta, 2005). It also deprives employees’ sense of meaningful existence by implying that he is unworthy to the organization. Accordingly, employees with low job security may lessen their sense of belongingness and their identification with the organization based on the norm of reciprocity.

Organizational identification, in turn, may affect OCBs from two aspects–cognitive and affective. From the cognitive standpoint, organizational identification reflects the employees’ self-categorization; when employees no longer regard themselves as part of the organization, they are less inclined to integrate their objectives with the organization’s goals, which may discourages their OCBs (Ashforth et al., 2008). Employees who highly identify with the organization, however, would see themselves as personifying the organization (Johnson and Ashforth, 2008), and dedicate to behaviors that are beneficial to the organization, compared to those who do not. From the affective angle, employees with a lower level of organizational identification may become psychologically separated from their organization, which may damage their motivation to invest more effort that benefits the organization (Roeck and Delobbe, 2012). Numerous empirical evidence also indicates that organizational identification is positively related with OCBs (Reisel et al., 2014; Ma et al., 2015; Stynen et al., 2013; Callea et al., 2016; Wu et al., 2016; Shoss, 2017). As such, we hypothesize that:

H3. Organizational identification mediates the relationship between job security and OCBs.

### Moderating Effect of Employment Status

We further propose that the negative relationship between job security and psychological contract breach is stronger for permanent employees than for contract employees. First, a psychological contract contains mutual expectancies between employers and employees regarding the obligations of both parties (Piccoli, and De Witte, 2015). Contract employees who sign a limited contract with an organization usually associate with the organization’s periphery, which, in turn, has been related with inferior job characteristics and organization status (Saloniemi et al., 2004). Therefore, contract employees, as compared with permanent employees, engage less in relational psychological contracting because they hold low expectancies regarding their job security than permanent employees. Second, the psychological contract theory also indicates that contract employees have a more transactional contract and have less relational expectation from the organization, whereas permanent employees hold a more relational psychological contract with the organization (Rousseau and Tijoriwala, 1998). This implies that contract employees focus on economic exchange, whereas permanent employees focus on economic and socio-emotional exchange with the organization. Permanent employees under a low job security may perceive that the organization has failed to fulfill their obligations by applying for a stable job in exchange for their effort (Vander Elst et al., 2016). However, contract employees who signed a limited contract with the organization do not interpret low job security as a breach of the implicit psychological contract between them and their employer because they are more focused on the economic social exchange with the organization. As a result, low job security is expected to be problematic for permanent employees, but not for temporaries. Thus, we hypothesize that permanent employees may more likely perceive psychological contract breach associated with low job security.

H4. Employment status moderates the relationship between job security and psychological contract breach, such that the negative relationship is stronger for permanent employees than contract employees.

Overall, we propose that a low job security will evoke a psychological contract breach mechanism with the organization and, thus, prevents employees under low job security from engaging in OCBs. We suggest that the effect of job security on a psychological contract breach is more prominent for permanent employees because they have more relational contract expectancies toward the organization. Our proposed model represents a first stage moderated mediation model. To test the moderated mediation effect in the model, we propose the following formal hypothesis:

H5. Employment status moderates the mediation effect of psychological contract breach on the relationship between job security and OCBs, such that the mediation effect is stronger for permanent employees than contract employees.

Social identity theory could also be used to investigate the different effects of job security on OCB across employment status. According to the social identity theory, the basic motive for identifying with a group is self-enhancement and uncertainty reduction motivation; that is, individual identify to provide the basis for thinking of themselves in a positive light and establish a stable future (Ashforth et al., 2008). Previous research has provided empirical findings that describe the different situations across employment status that suggest that relative to permanent employees, contract employees experience inferior job status and higher levels of job instability (Broschak and Davis-Blake, 2006; Cappelli and Keller, 2013; Camuffo and Stefano, 2016). Social identity theory indicates that employees use groups as sources of information about themselves (Fuller et al., 2016). This imply that employees may use the status of their organization to assist them in assessing their self-worth and thinking of themselves in a positive light by seeking organizational identification. Therefore, for contract employees, the basic needs for establishing a positive self-esteem and reducing uncertain by identifying with the organization are stronger, compared to permanent employees, due to their inferior situation, which results in a stronger positive relationship between job security and organizational identification.

However, the effect of job security on organizational identification may be less influential among permanent employees because, with a more predictable job future, they are less likely to rely on the quality of job security as an identification cue to satisfy the uncertainty reduction and self-enhancement motives. Whereas, for contract employees, job security provides them crucial assurances and resources regarding their job future and their organizational membership, which is more critical for them and propels them to develop their organizational identification. Therefore, we hypothesize that:

H6. Employment status moderate the relationship between job security and organizational identification, such that the negative relationship is stronger for contract employees than permanent employees.

Overall, we propose that a low job security will less evoke a social identity mechanism with the organization and, thus, prevent employees from engaging in OCBs. We suggest that the effect of job security on organizational identification is more prominent for contract employees because they have a stronger motivation for uncertainty reduction and self-enhancement than permanent employees. Our proposed model represents a first stage moderated mediation model. To test the moderated mediation effect in the model, we propose the following formal hypothesis:

H7. Employment status moderate the mediation effect of organizational identification on the relationship between job security and OCBs, such that the mediation effect is stronger for contract employees than permanent employees.

## Research Methods

### Data and Sample

We collected data from employees of a state-owned airline company in China. The participants in our research include managers, professional, service, and support staff. Separate questionnaires were developed and administered to 235 employees and 70 direct supervisors. Each employee completed a survey containing items tapping job security, psychological contract breach, organizational identification, employment status, and individual demographic information. One month later, their immediate supervisor rated the employees’ OCBs. In total, data were collected from 211 employees and their 61 supervisors, a response rate of 89.8% for employees and 87.1% for supervisors. In the process of data collection, uncompleted responses were excluded, 211 subordinate-supervisor dyads were obtained with a 97.23% effective rate. Of the 211 respondents, 102 were contract employee (48.3%) and 109 (51.7%) were permanent employees, more than half of the respondent (57.35%) were female, 46.1% of the employees were younger than 30 years old and had completed university degrees.

### Measures

For all measures, respondents rated the items on a five-point scale, ranging from 1 = strongly disagree to 5 = strongly agree. The questionnaires were presented in Chinese language.

#### Job Security

Job security was measured with a six-item scales developed by Gong and Chang (2009). A sample item was “Employees in our firm can expect to stay for as long as they wish.” The Cronbach α coefficient for the scale was 0.86.

#### Organizational Identification

We assessed organizational identification using a five-item scale proposed by Smidts et al. (2001). The questionnaire was filled by employees. A sample item was “I feel proud to work for my company.” The reliability was 0.84.

#### Psychological Contract Breach

We adapted Tekleab et al.’s (2005) three-item scale to measure psychological contract breach. A sample item was “The company has repeatedly failed to meet its obligations to me.” The Cronbach α coefficient for the scale was 0.83.

#### Organizational Citizenship Behaviors

Organizational citizenship behaviors were assessed with Farh et al.’s (1997) four-item scale and reported by employees’ supervisors. A sample item was “Willing to cover work assignments for colleagues when needed.” The Cronbach α coefficient for the scale was 0.93.

#### Employment Status

Employment status was reported by the supervisors; permanent employees was coded as 0, and contract employees was coded as 1.

#### Control Variables

Several demographic characteristics of employees were controlled in our analysis. Employees reported their gender (coded 1 = male; 2 = female), age (coded 1 < 30 years old; 2 = 30–39 years old; 3 = 40–49 years old; 4 ≥ 50 years old), education experience (coded 1 = middle school or below; 2 = high school or secondary school; 3 = junior college; 4 = university; 5 = post-graduate or above), and tenure (coded 1 < 2 years; 2 = 2–3 years; 3 = 4–5 years; 4 = 6–7 years; 5 ≥ 8 years).

### Analysis

Following Anderson and Gerbing (1988) instruction, the analysis had two steps: first, the measurement model was tested by using confirmatory factor analysis (CFA) to examine the divergent validity. The measurement model was first fitted to the data separately for temporary and permanent workers, which is critical to establish discriminant validity. Second, we compared the fit index among several competing structural model; then, the best fitted model was chosen for subsequent multiple group analyses. Data were analyzed using the Amos 24.0 and SPSS software package.

Specifically, we first test the divergent validity of variables, including job security, organizational identification, psychological contract breach, and OCB separately for contract and permanent employees, before testing it for all 211 employees. If the measurement model had a good fit, we then used the maximum-likelihood method to examine the structural model to assess whether it is fitted to the data. Among the different competing models, the best fitted was selected for further analyses. A number of goodness-of-fit indices was used to examine the model fit: Chi-square/degree of freedom (χ^2^/df), root-mean-square residual (RMR), comparative fit index (CFI), incremental fit index (IFI), root-mean-square error of approximation (RMSEA), parsimony normed fit index (PNFI), and parsimony comparative fit index (PCFI). Lastly, multiple-group analysis was applied to compare the path coefficients of contract and permanent employees, and judge whether significant differences exist between the two groups.

## Results

Table 1 presents the respective means and standard deviations for permanent and contract employees, and correlations of all samples. All the internal consistencies were satisfactory. Job security was positively correlated with organizational identification, OCB, and negatively related with psychological contract breach; the permanent employees (*M* = 3.78) tend to gain more job security than contract employees (*M* = 3.24). Except for psychological contract breach, other key variables show a significantly difference between permanent and contract employees.

**TABLE 1:** Means, standard deviations, correlations of variables.

	**Temporary employees**		**Permanent employees**									
	**M**	**SD**	**M**	**SD**	**1**	**2**	**3**	**4**	**5**	**6**	**7**	**8**
1 Sex	1.54	0.50	1.61	0.49								
2 Age	1.42	0.60	1.91	0.76	−0.14*							
3 Education	2.76	0.95	3.20	0.76	0.33**	–0.02						
4 tenure	2.76	1.18	4.28	1.16	0.08	0.49**	0.11					
5 JS	3.24	1.11	3.78	1.04	–0.01	0.16*	0.01	0.13	(0.86)			
6 OI	3.54	0.68	3.72	0.56	–0.01	0.12	–0.04	0.01	0.44**	(0.84)		
7 PCB	2.48	0.82	2.36	0.70	–0.05	–0.08	–0.02	–0.03	−0.34**	−0.64**	(0.83)	
8 OCB	3.94	0.77	4.17	0.61	0.09	0.04	0.23**	0.09	0.27**	0.54**	−0.30**	(0.93)

*N* (Temporary employees) = 102; *N* (Permanent employees) = 109. JS, job security; OI, organizational identification; PCB, psychological contract breach; OCB, organizational citizenship behavior. Reliabilities are listed in parentheses. *p < 0.05; **p < 0.01 (two tailed).

### The Measurement Model

We first texted a measurement model that estimated all focal latent constructs by having their respective measurement items load on their corresponding latent factors as indicators. Obtaining a satisfactory fit for the measurement model is critical to establish discriminant validity and to inspect risks associated with common method variance (Podsakoff et al., 2003). For the variables rated by contract employees (i.e., job security, organizational identification, psychological contract breach, OCB), the CFA results showed that the hypothesized four-factor model yielded a satisfactory fit [χ^2^(129) = 239.315, CFI = 0.922, RMR = 0.044, RMSEA = 0.091, IFI = 0.923, TLI = 0.907], than a three-factor model (i.e., job security, psychological contract breach, OCB) as a combined factor [χ^2^(132) = 307.48, CFI = 0.88, RMR = 0.06, RMSEA = 0.11, IFI = 0.88, TLI = 0.86], a two-factor model (i.e., job security, organizational identification) as a combined factor [χ^2^(134) = 501.78, CFI = 0.74, RMR = 0.10, RMSEA = 0.16, IFI = 0.74, TLI = 0.70], and a one-factor model, with all three variables as a combined factor [χ^2^(135) = 584.51, CFI = 0.68, RMR = 0.10, RMSEA = 0.18, IFI = 0.69, TLI = 0.64].

For the permanent employees, the CFA results showed that the hypothesized four-factor model (i.e., job security, organizational identification, psychological contract breach, OCB) yielded a satisfactory fit [χ^2^(129) = 268.01, CFI = 0.92, RMR = 0.06, RMSEA = 0.05, IFI = 0.92, TLI = 0.90] than a three-factor model (i.e., job security, psychological contract breach, OCB) as a combined factor [χ^2^(132) = 715.24, CFI = 0.66, RMR = 0.11, RMSEA = 0.20, IFI = 0.66, TLI = 0.60], a two-factor model (i.e., job security, organizational identification) as a combined factor [χ^2^(134) = 779.20, CFI = 0.62, RMR = 0.09, RMSEA = 0.21, IFI = 0.63, TLI = 0.57], or a one-factor model, with all three variables as a combined factor [χ^2^(135) = 1,233.57, CFI = 0.36, RMR = 0.13, RMSEA = 0.27, IFI = 0.36, TLI = 0.27]. The CFA provided a support for the discriminant validity of measurement.

### The Structural Model

As seen in Table 2, the baseline model (model 1) included a good fit to the data: χ^2^(258) = 494.74, RMR = 0.05, CFI = 0.91, IFI = 0.91, and RMSEA = 0.07. We then added some constraints to the model. First, we restricted the equality of variance in model 2, yielding a fit [χ^2^(280) = 657.46, RMR = 0.07, CFI = 0.85, IFI = 0.85, and RMSEA = 0.09]; and then restricted the equality of path coefficient in model 3, revealing that χ^2^(272) = 517.62, RMR = 0.05, CFI = 0.90, IFI = 0.90, and RMSEA = 0.07. Lastly, in model 4, we restricted both variance and path coefficient, which did not yield an adequate fit: χ^2^(294) = 678.91, RMR = 0.10, CFI = 0.85, IFI = 0.85, and RMSEA = 0.08. Overall, these restricted models did not yield an adequate fit.

**TABLE 2:** Fit Indices of structural models.

**Multiple Group Model**	**χ^2^/df**	**RMR**	**CFI**	**IFI**	**RMSEA**
Model 1 (baseline model)	1.92	0.05	0.91	0.91	0.07
Model 2 (variance be equal)	2.35	0.07	0.85	0.85	0.09
Model 3 (path coefficient be equal)	1.90	0.05	0.90	0.90	0.07
Model 4 (variance and path coefficient be equal)	2.31	0.10	0.85	0.85	0.08

**N* = 211. RMR, root mean square residual; CFI, comparative fit index; IFI, incremental fit index; RMSEA, Root Mean Square Error of Approximation. All models were compared with Model 1.*

### Hypothesis Test

**FIGURE 2:**
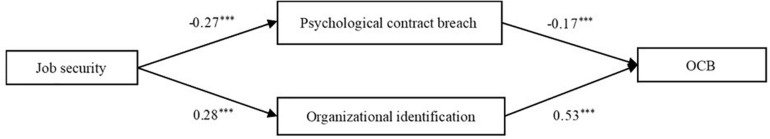
Final model estimators for total workers. ***p < 0.001.

As observed in Table 3, after controlling the demographic characteristics, the positive association between job security and OCB was significant (β = 0.305, *p* < 0.001), which supported Hypothesis 1. Following Preacher and Hayes’ (2008) suggestion, investigating multiple mediation should involves two parts: (1) investigating the total indirect effect; and (2) testing hypotheses regarding individual mediators, that is, investigating the indirect effects associated with each mediator. We use this macro because it allows us to compare the strengths of two indirect effects in order to explore which underlying theory should be given more credence, namely, a contrast test. Therefore, as shown in Table 4 and Figure 2, we first found that job security was positively related to OCB through organizational identification and psychological contract breach: the total indirect effect was 0.20 (*p* < 0.001). Specifically, the relationship between job security and psychological contract breach was negative (−0.27, *p* < 0.001). Job security also had a positive relationship with organizational identification (0.28, *p* < 0.001). In turn, psychological contract breach was positively related to OCB (−0.17, *p* < 0.001) and organizational identification was positively related to OCB (0.53, *p* < 0.001).

**TABLE 3:** Main effect of job security on OCB.

	**OCB**			
	**Model 1**	**Model 2**	**Model 3**	**Model 4**
Gender	0.03	0.03	0.01	0.01
Age	–0.01	–0.03	–0.09	–0.04
Education	0.21**	0.20***	0.24***	0.21**
tenure	0.08	0.06	0.10	0.07
JS		0.31***	0.08	0.23**
OI			0.48***	
PCB				−0.19**
*R*^2^	0.04	0.13	0.31	0.16
*F*	3.14*	7.17***	16.49***	7.48***

*N* = 211. JS, job security; OI, organizational identification; PCB, psychological contract breach; OCB, organizational citizenship behavior. *p < 0.05; **p < 0.01; ***p < 0.001 (two tailed).

**TABLE 4:** Results of the analyses for the multiple mediation model using the SPSS-macro of Preacher and Hayes (2008).

	**OCB**			
	**Coefficient**	**SE**	***P***	**Bootstrap 95% CI**
IV to mediators				
Psychological contract breach	–0.27	0.05	<0.001	
Organizational identification	0.28	0.04	<00.001	
Direct effect of mediators to DV				
Psychological contract breach	–0.17	0.06	<00.001	
Organizational identification	0.53	0.72	<0.001	
Total effect of IV on DV	0.20	0.04	<0.001	[0.12; 0.29]
Direct effect of IV on DV	0.06	0.04	>0.05	[−0.02; 0.15]
Total indirect effect of IV on DV through proposed mediators	0.14	0.04	<0.001	[0.08; 0.23]
Psychological contract breach	–0.02	0.03	>0.05	[−0.08; 0.02]
Organizational identification	0.16	0.05	<0.001	[0.09; 0.29]
Contrast test Psychological contract breach vs. organizational identification	0.27	0.09	<0.05	[0.11; 0.46]

*IV, independent variable, that is, job security; DV, dependent variable, that is OCB. If zero is not included in the interval, the effect is significant.*

Furthermore, the results of the single and multiple mediator tests are displayed in Table 4, respectively. The results of the single mediator analysis with psychological contract breach as the mediator showed that psychological contract breach mediated the relationship between job security and OCB. This result supported Hypothesis 2, considering the indirect effect (−0.27, *p* < 0.001). Next, the single mediator analysis with organizational identification as the mediator supported that organizational identification mediated the relationship between job security and OCB (indirect effect 0.28, *p* < 0.001), which is in line with Hypothesis 3. We further examined the mediating role of psychological contract breach and organizational identification simultaneously using OLS analysis. The mediating mechanism of psychological contract breach between job security and OCB is not significant when considering the two mediators. The test of the difference between the indirect effects of both mediators also indicated that organizational identification was the most important factor in mediating the impact of job security on OCB (see the contrast result in Table 4).

We used a multiple-group path model to determine why there was a significant difference between contract and permanent employees in terms of the effect of job security on OCB. The baseline model showed a fit [χ^2^(262) = 575.56, *p* < 0.01], and the model with a restricted path coefficient to equality yielded a fit [χ^2^(280) = 630.15, *p* < 0.01]. Therefore, the chi-square difference between these two models was significant (Δχ^2^ = 54.59, *p* < 0.01); that is, the paths exhibited differences between two types of workers in our model, and can be compared. We used critical ratios for differences between parameters (CRD) to judge if these paths have significant differences. An absolute value of CRD greater than 1.96 indicates estimators are significantly different; if below 1.96, the difference between estimators can be regarded as significantly equal to 0 (Hu and Bentler, 1999).

**FIGURE 3:**
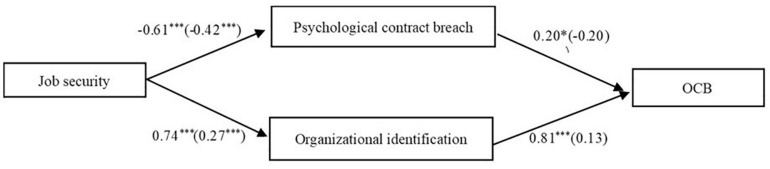
Final model estimators for contract and permanent employees (in parentheses). *p < 0.05; ***p < 0.001.

Hypothesis 4 stated that employment status moderates the relationship between job security and psychological contract breach, such that the negative relationship is stronger for permanent employees than contract employees. In this study, the estimators between job security and psychological contract breach showed a CRD value of 1.39, indicating the estimators between job security and psychological contract breach in permanent employees has no significant difference compared with those in contract employees. The results did not support Hypothesis 4. And Hypothesis 5 stated that employment status moderates the mediation effect of psychological contract breach on the relationship between job security and OCBs, such that the mediation effect is stronger for permanent employees than contract employees. As there is no significant difference between job security and psychological contract breach across employment status, this Hypothesis 5 was also not supported. However, our results revealed that the CRD value between psychological contract breach and OCB (−2.81) was above 1.96, manifesting the divergent relationship between psychological contract breach and OCB in permanent employees (β = −0. 20, *p* > 0.05) and contract employees (β = 0.20, *p* < 0.05). And Figure 3 shows the results for temporary and permanent workers. The results indicate that job security is negatively related with psychological contract breach for both temporary (β = −0.61, *p* < 0.001) and permanent employees (β = −0.42, *p* < 0.001). In turn, psychological contract breach is positively related with OCB for temporary workers (β = 0.20, *p* < 0.05). This path was not significant for permanent workers (β = −0.20, *p* > 0.05).

Hypothesis 6 stated that employment status moderates the relationship between job security and organizational identification, such that the negative relationship is stronger for contract employees than permanent employees. For the estimators between job security and organizational identification (−2.93), the CRD value was above 1.96, indicating that the estimators between job security and organizational identification for permanent employees was significantly different from contract employees. For contract employees (β = 0.74, *p* < 0.001), the job security-organizational identification was significantly stronger, compared to permanent employees (β = 0.27, *p* < 0.001), which supported Hypothesis 6. Hypothesis 7 stated that employment status moderates the mediation effect of organizational identification on the relationship between job security and OCBs, such that the mediation effect is stronger for contract employees than permanent employees. The results demonstrated that the effect of organizational identification on OCB (CRD value: −5.18) in permanent employees (β = 0.81, *p* > 0.05) was significantly higher than contract employees (β = 0.13, *p* < 0.05), which supported Hypothesis 7.

## Discussion

The first aim of the present article was to understand the reasons of discretionary OCBs resulting from subjective job security, extending knowledge on theoretical explanations. However, research on why job security influence individuals’ OCB is scarce and is mainly focused on a single explanation, neglecting the possible interplay of manifold mediators in the same relationship (De Witte et al., 2016). For example, Callea et al. (2016) research focused on the mediating mechanism of organizational identification between job security and OCB. Another aim of the present study was to understand the potential differences in the OCB respond to subjective job security among permanent and contract workers from the perspective of social identity and social exchange in Chinese hybrid employments. The increasing use of contract employees necessitates the need to consider the prevailing hybrid employment contexts in exploring the equivocal relationship between job security and OCBs. Although previous research has demonstrated that job security can have different effects on OCBs across employment status (Liu et al., 2017), no research has explained the reason for these effects. In particular, we assumed psychological contract breach and organizational identity as two different mechanisms to delineate the effect of job security on OCB for all employees and across employment status. We also investigated whether psychological contract breach or organizational identification plays a leading role in how job security affects employees OCBs.

Using 211 dyads of employees and supervisors, the results indicated that job security could influence employees’ psychological contract breach and organizational identification, in turn, impacting their OCBs, which replicated earlier studies that revealed the negative influence exerted by low level job security on OCBs (Ma et al., 2015; Piccoli et al., 2017). For all employees sampled, organizational identification performed a bigger mediating function than psychological contract breach in the relationship between job security and OCBs. This may be attributed to the fact that incentives-based relationships between employees and their organization proceed more from employees’ inner identification to the organization than simply from reciprocity of behavior.

Results of this study support using an identification perspective to understand the different association between job security and OCB among contract and permanent worker. We found that for contract employee, the associations between job security and organizational identification as well as organizational identification and OCB is stronger than permanent employees. This can be explained by the social identity theory, individuals tend to form organizational identification to uncertainty reduction motivation; that is, individuals identify to provide the basis for thinking of themselves in a positive light and establish a stable future (Ashforth et al., 2008). Contract employee usually signed a limited contract and in a relative low status situation, the basic needs for establishing a positive self-esteem and reducing uncertain by identifying with the organization are stronger than permanent employees and thus they are more likely to engage in OCB. Besides, our finding also suggests that there is no significant relationship between permanent workers’ organizational identification and their OCB (β = 0.13, *p* > 0.05). This finding may be explained by the employees in Chinese state-owned company who tend to view job security for granted and may not engage in OCB to reciprocate their company. Even though they identify their organization, they are less likely engage in OCB due to the motivation of job security is not important to them.

Contrary to expectations, the results also showed that there is no significant difference between job security and psychological contract breach among contract and permanent workers. This may be because everyone is pursuing stable and sustainable working conditions, whether it is permanent or contract worker. Therefore, in the case of a low level of job security, there will be no significant difference responding to job security with psychological contract breach across employment status.

Another surprising result were findings the association between psychological contract breach and OCB is non-significant among permanent workers (β = −0.20, *p* > 0.05), and positive among contract workers (β = 0.20, *p* < 0.05). These ties are not consistent with previous studies (Lo Presti et al., 2019). This may be explained by the special properties of Chinese state-owned employment context in which permanent employees cannot be easily dismissed; the stimulus for a decreased psychological contract breach has not been sufficient to promote their conduction on the OCBs. On the other hand, permanent employees in Chinese state-owned enterprise who tend to possess high levels of human capital than permanent employees and thus less likely to be influenced by psychological contract breach resulted from low levels of job security. While for contract worker, even if they experience a lower level of job security, leading to the breach of their psychological contract, they still participate in OCB. The results can be explained by the social exchange theory (Cropanzano, 2005), which suggests that the organization could provide inducement to employees by means of providing an opportunity to join and stay in exchange for their contribution to the organization. The lure of stable work works well for contract employees, compared to permanent employees, as they do not perceive having a formal status in the organization in their job stability situation. Thus, contract employees would be empowered to enhance their OCBs, even with a perceived chance of a psychological contract breach (β = 0.20, *p* < 0.05), which increases their likelihood of joining the organization, as they expect to obtain a permanent contract (De Cuyper et al., 2014). By contrast, higher job security would have a less effect on the OCBs of permanent employees through psychological contract breach in Chinese state-own organizations. Therefore, for contract employees, OCB can serve as an instrumental means to gaining favor and recognition with the employer, and, thus, job security can enhance their OCB through preventing them from suffering a psychological contract breach. On the other hand, even though they are confronted with psychological contract breach, they are unlikely to decrease their OCB because they look forward to obtain a permanent position in the organization.

### Theoretical Implications

First, we fill the gap in research on behavioral responses to job security, using social identify and psychological contract breach perspective to provide a more detailed explanation of the process of job security in shaping employees’ OCBs across employment status. Although previous research has found a negative relation between job security and employees’ behavior through organizational identity or psychological contract breach (Ma et al., 2015; Piccoli, and De Witte, 2015), these studies have neglected the possible interplay of multiple mediators in the job security-OCB relationship. Our research provides a more comprehensive explanation for the behavioral consequences associated with job security by considering social identity and psychological contract breach. Extending these models, our research unpacked not just the coexistence of such mechanisms in the job security domain, but their relative significance in the presence of each other. Our findings revealed that organizational identification has a greater mediating effect than psychological contract on the interplay of job security and OCBs, shedding new lights on how job security affects employee behavior on a broader perspective.

Second, we also contribute to the job security literature by examining the mechanisms that interpret the reasons for the different relationship between job security and OCB among permanent and contract employees. We build on findings that suggest employment status differences are likely to explain the inconsistent finding of the effect of job security on OCB by further investigating the differences from psychological contract breach and social identification perspectives (Liu et al., 2017). Our study reveals that job security could promote contract employees’ OCBs through the mechanism of social identity. We showed that job security has stronger effects on organizational identification, and leads to a stronger motivation for performing OCBs in contract employees compared to permanent employees. This extension is meaningful because it suggests that job security can shape different levels of organizational identification among employment status and, thus, influence the intrinsic force driving OCBs.

Third, we also broadened previous research by demonstrating that for permanent employees, organizational identification and psychological contract breach resulting from job security cannot sufficiently propel them to perform OCBs in Chinese state-owned contexts, which calls for more explanations and comments. This finding is distinct from previous research (Callea et al., 2016; Vander Elst et al., 2016), and also unexpected from the standpoint of social identity (once employee with high level organizational identification, they are more likely to reciprocate their organization with OCBs). Further, the observation also challenges the psychological contract breach perspective (permanent employee would balance the exchange relationship with organization by reducing their OCBs when psychological contract breach is concerned), supplementing the job security research in the Chinese context. This may be due to the fact that although the organization provides employees with job security, and induces employees to form an organizational identity, it does not inspire individuals to perform more OCBs because employees are guaranteed a lifetime contract. For these employees, there is no need to make additional efforts to obtain the sustainability of the work, and their motivation for engaging in OCB is reduced.

Fourth, previous research has not clarified the relationship between job security and organizational identification among permanent and contract employees (De Cuyper et al., 2009). Our results addressed this gap by revealing that the relationship between job security and organizational identification is more positive for contract employees than for permanent employees. The finding of a more positive relationship between job security and organizational identification furthermore concurs with our interpretation that contract workers have more self-enhancement and uncertainty reduction needs than permanent employees–may be more encouraged to cultivate organizational identification when the employer provides a secured job for them.

### Practical Implications

The current findings also have two important practical implications. First, our results provide some evidence that employees with less job security perform discretionary OCBs according to their perceptions of a psychological contract breach and organizational identification. Therefore, organizations may need to pay attention to the promise of psychological contract and fulfill their obligations to these employees. For example, managers should maintain proactive communication with employees to elaborate their psychological contract and predict employees’ attitudes toward the organization, which may contribute to the lowering of the employees’ perception of a psychological contract breach. Most importantly, organizational identification exerts a more leading influence on the effect of job security on OCBs than a psychological contract breach. Practitioners may use this knowledge to forestall low levels of job security that results in fewer OCBs. In this respect, organizations may need to exert more effort in realizing the socialization process of employees and cultivate all employees’ identity toward the organization.

Our results also highlight that organizational identification related with job security is not enough to elicit permanent employees’ OCBs, but that contract employees’ job security is more likely to encourage the formation of organizational identification and restrain the negative effect of psychological contract breach. These results imply that managers should design different organizational interventions to motivate employees of different status. What’s more, organization should pay more attention to improve the work condition of temporary workers, such as providing a promotion opportunity for contract workers as well as more organizational support to them. In the employee-organization relationship, reciprocity principle play a leading role in the process of exchange, and thus, the organization should exhibit as much humanity concern for contract employee to motivate their OCB. Previous research also suggested that temp-to permanent strategy can improve the organizational outcomes of workforce blending (Fisher and Connelly, 2017).

### Limitations and Future Research

There are some limitations in our research. First, the data were collected merely from one company in China, limiting the generalizability of the results. We encourage further research to draw on various firms and industries to increase the generalizability of the results. Second, our results showed job security affected contract employees’ OCBs through the mechanisms of psychological contract and organizational identification, but these two mechanisms were not operational in permanent employees. This may be resulted from our limited samples. Future research could replicate this study to reveal this “black box” to better understand the effects of job security on permanent employees’ OCBs in the Chinese context by a larger number of permanent and contract workers participants. Besides, due to the main purpose of our research is to explore the different response across employment status in the hybrid employment context, our research did not consider the chained intermediary mechanism of organizational identification and psychological contract breach between job security and OCB. Since both organizational identification and psychological contract breach represent different state employee-organizational relationships, future research can further explore the chained intermediary mechanisms of organizational identity and psychological contract breakdown between job security and OCB. Finally, some indicators of our research sample model are not perfect, such as the RMSEA and RMR are too high, and it is more perfect if these indices are lower than 0.05.

## Data Availability Statement

The raw data supporting the conclusions of this article will be made available by the authors, without undue reservation.

## Ethics Statement

Ethical review and approval was not required for the study on human participants in accordance with the local legislation and institutional requirements. The patients/participants provided their written informed consent to participate in this study.

## Author Contributions

WL is a Ph.D. student in human resource management. She is responsible for conception and design of the study, analysis and interpretation of the data, drafting of the manuscript. XL is a assistant professor in human resource management. She is responsible for design of the survey, revising of the manuscript. SL is a professor with a doctorate in human resource management. He is responsible for conception and design of the study and collection of the data. CQ is a Ph.D. student in human resource management. She is responsible for revising of the manuscript and improving of the English writing. All authors contributed to the article and approved the submitted version.

## Conflict of Interest

The authors declare that the research was conducted in the absence of any commercial or financial relationships that could be construed as a potential conflict of interest.
